# IL-33 released by alum is responsible for early cytokine production and has adjuvant properties

**DOI:** 10.1038/srep13146

**Published:** 2015-08-14

**Authors:** William A. Rose II, Angela J. Okragly, Chetan N. Patel, Robert J. Benschop

**Affiliations:** 1 Biotechnology Discovery Research, Lilly Research Laboratories, Eli Lilly and Company, Indianapolis, IN, 46285

## Abstract

Human vaccines have used aluminium-based adjuvants (alum) for >80 years despite incomplete understanding of how alum enhances the immune response. Alum can induce the release of endogenous danger signals via cellular necrosis which elicits inflammation-associated cytokines resulting in humoral immunity. IL-33 is proposed to be one such danger signal that is released from necrotic cells. Therefore, we investigated whether there is a role for IL-33 in the adjuvant activity of alum. We show that alum-induced cellular necrosis results in elevated levels of IL-33 following injection *in vivo*. Alum and IL-33 induce similar increases in IL-5, KC, MCP-1, MIP-1α and MIP-1β; many of which are dependent on IL-33 as shown in IL-33 knockout mice or by using an IL-33-neutralizing recombinant ST2 receptor. Furthermore, IL-33 itself functions as an adjuvant that, while only inducing a marginal primary response, facilitates a robust secondary response comparable to that observed with alum. However, IL-33 is not absolutely required for alum-induced antibody responses since alum mediates similar humoral responses in IL-33 knockout and wild-type mice. Our results provide novel insights into the mechanism of action behind alum-induced cytokine responses and show that IL-33 is sufficient to provide a robust secondary antibody response independently of alum.

Adjuvants are used to enhance the efficacy of vaccines by boosting the immune response through different mechanisms of action^1^,^2^. Understanding how an adjuvant activates the immune response is important for rational vaccine design to tailor the response based on the antigen and type of immunity necessary to protect against infection. In 1926, Glenny *et al.* showed that an aluminium-based adjuvant (alum) enhanced the antibody response to diphtheria toxin in guinea pigs^3^ and a similar enhancing effect was observed in humans in 1932^4^. While aluminium-based adjuvants are the main immunostimulants used in human vaccines^1^,^2^,^5^, their mechanism(s) of action remain poorly understood. It was originally proposed that alum acted as a “depot” and allowed for slow, long-term release of the antigen into the body^1^,^3^. However, recent data showed that eliminating the potential depot function by inhibition or removal of alum-induced nodules did not impact the adjuvant activity of alum^6^,^7^.

Recently, several new mechanisms of actions were proposed to explain the adjuvant activity of alum. It was shown that alum drives a T_H_2-biased immune response mediated through production of IL-5, IL-13 and other inflammation-associated cytokines^8^,^9^,^10^,^11^. These cytokines in combination with neutrophils, monocytes, eosinophils and mast cells recruited to the alum injection site induced B cell proliferation and activation resulting in the production of antigen-specific antibodies in mice^10^,^12^,^13^. Another proposed mechanism of alum-induced adjuvant activity involves the release of endogenous danger signals or “alarmins” via alum-mediated localized cellular damage^14^. These alarmins, including uric acid, can directly stimulate the inflammasome via NLRP3 resulting in activation of a humoral immune response^15^,^16^,^17^,^18^. It also was shown that alum-induced cytotoxicity resulted in the release of host DNA that partially mediated the adjuvant activity of alum by enhancing antigen presentation^19^,^20^.

Cellular damage caused by alum also could induce the release of intracellular cytokines like IL-1α, HMGB-1 and IL-33^21^,^22^. IL-33 is a member of the IL-1 family of cytokines and is primarily present in fibroblasts, epithelial cells and endothelial cells^21^,^23^,^24^. Full length IL-33 contains an N-terminal chromatin binding domain that results in nuclear localization^25^,^26^. A proposed mechanism of IL-33 release from the nuclear compartment involves cellular necrosis and subsequent enzymatic cleavage of the N-terminal chromatin binding domain at several different sites via neutrophil and mast cell-released inflammatory proteases^22^,^27^,^28^. Both the full length and the cleaved “mature” forms of IL-33 bind to ST2, a receptor commonly expressed on immune and structural cells^23^,^27^,^28^,^29^. While mature IL-33 forms exhibit greater *in vitro* and *in vivo* activity than full length IL-33, all forms induce T_H_2 and inflammation-associated cytokine release following binding to the ST2 receptor^27^,^28^,^29^.

In this study, we investigated whether there is a role for IL-33 in alum-induced immune responses. We show that alum caused the release of IL-33 via the induction of cellular necrosis. The production of a T_H_2- and inflammation-associated cytokine profile induced by alum was similar to that observed following IL-33 injection and neutralization of IL-33 eliminated the alum-induced cytokine production. Moreover, administration of IL-33 with antigen resulted in the induction of antigen-specific antibody responses, indicating that IL-33 itself has adjuvant activity. However, the IL-33-mediated primary antibody response kinetics differed from that observed with alum, and lack of IL-33 did not alter alum-induced humoral responses. Collectively, these results provide novel insights into the mechanism of action behind alum-induced cytokine responses and show that IL-33 alone is sufficient to provide a robust secondary antibody response.

## Results

### Alum induces release of IL-33 via cellular necrosis

**Figure 1:**
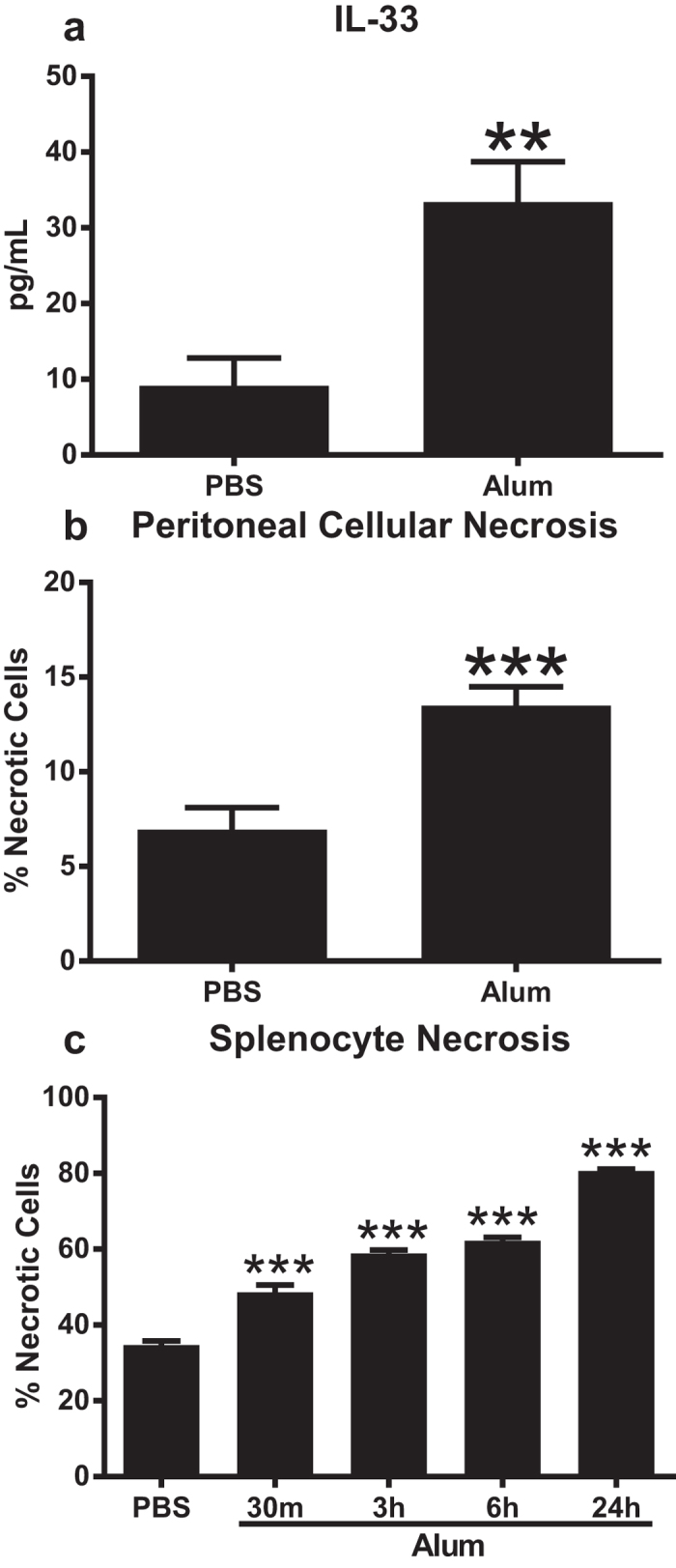
Alum induces release of IL-33 via cellular necrosis. C57BL/6 mice (n = 5–8 mice/group) were injected i.p. with PBS or alum mixed with PBS at 1:2 ratio and peritoneal lavages were collected thirty minutes later. Peritoneal lavages were analyzed for IL-33 levels via ELISA (**a**) and percentage of necrotic cells using ViaCount solution which contains two proprietary DNA binding dyes to differentiate between viable, apoptotic, and dead cells (**b**). Data are combined from two independent experiments. **p < 0.01 PBS compared to alum injected groups (Two-tailed unpaired Student t-test). (**c**) Splenocytes isolated from naïve C57BL/6 mice were cultured with PBS (24 h) or alum mixed with PBS at a 1:2 ratio (n = 3 wells/group) for the indicated times then cellular necrosis was quantified as in (**b**). Data are representative of three independent experiments. ***p < 0.01 PBS compared to alum treated groups (One-way ANOVA with Dunnett’s test).

It has been reported that alum induces cellular necrosis and release of DNA following intraperitoneal (i.p.) injection^19^, and IL-33 is proposed to be released from necrotic cells as well^22^,^27^,^28^. To show a potential direct link between alum-induced cellular necrosis and release of IL-33, we quantified IL-33 in the peritoneal cavity thirty minutes after i.p. injection of alum in mice. IL-33 levels were significantly increased in alum injected wild-type (WT) mice compared to PBS (Fig. 1a). IL-33 was not detected in IL-33 knockout (KO) mice injected with alum or PBS (data not shown). The percentage of necrotic cells isolated from the peritoneal cavity was significantly increased in the alum injected WT mice compared to PBS treated mice (Fig. 1b). To confirm these results and more directly quantify alum-induced cellular necrosis, splenocytes were isolated from naïve WT mice and cultured with alum or PBS for selected times. As shown in Fig. 1c, alum induced a significant increase in cellular necrosis over time. IL-33 levels from *in vitro* cultures were below the limit of detection for the IL-33 ELISA (data not shown). Thus, injection of alum into the peritoneal cavity resulted in rapid cellular necrosis and release of IL-33.

### Alum and IL-33 elicit similar cytokine profiles

**Figure 2:**
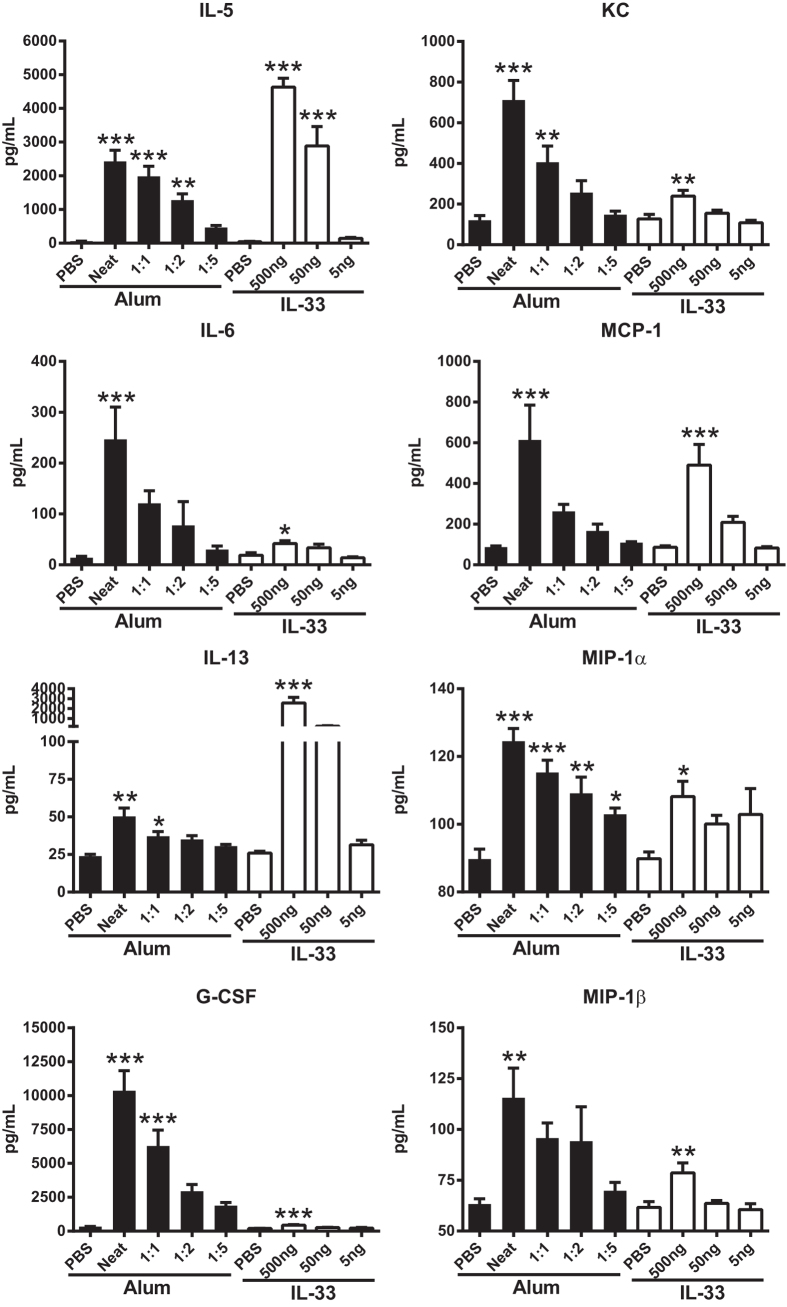
Alum and IL-33 induce the production of T_H_2 and inflammation-associated cytokines. C57BL/6 mice were injected i.p. with different concentrations of alum (n = 9–10 mice/group; black bars) or IL-33 (n = 5 mice/group; white bars) and serum was collected six hours later for multiplex cytokine analysis. Data are representative of at least two independent experiments. *p < 0.05 and **p < 0.01 and ***p < 0.001 PBS compared to alum injected groups or PBS compared to IL-33 injected groups (One-way ANOVA with Dunnett’s test).

**Figure 3:**
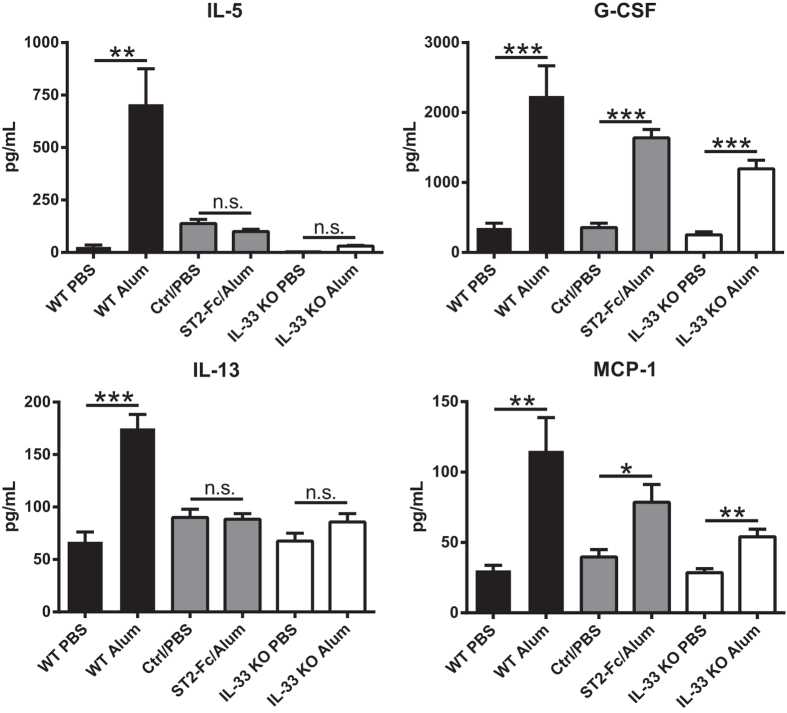
IL-33 drives alum-induced cytokine production. WT (black bars) and IL-33 KO (white bars) mice (n = 10–15 mice/group) were injected i.p. with PBS or alum mixed with PBS at 1:2 ratio (Alum). Additionally, WT mice (n = 10 mice/group) were injected i.p. with mouse IgG1 isotype control antibody (Ctrl) or ST2-Fc (grey bars) one hour prior to i.p. injection of PBS or alum mixed with PBS at 1:2 ratio. Serum was collected from all groups six hours later for multiplex cytokine analysis. Data are representative of at least two independent experiments. *p < 0.05 and **p < 0.01 and ***p < 0.001 PBS compared to alum (Two-tailed unpaired Student t-test, n.s. = not significant).

It is known that i.p. injection of alum or IL-33 results in increased serum cytokine levels^8^,^9^,^10^,^11^,^23^. Based on the observed release of IL-33 after i.p. injection of alum (Fig. 1a), we compared the cytokine response elicited by alum or IL-33. Serum was obtained from mice six hours post-injection of selected alum or IL-33 concentrations i.p. and cytokines were quantified using a multiplex ELISA. Both alum and IL-33 induced significant dose-dependent increases in IL-5, IL-6, IL-13, G-CSF, KC, MCP-1, MIP-1α and MIP-1β (Fig. 2). Interestingly, IL-33 induced a much more potent IL-13 response while alum showed a greater release of G-CSF. No significant induction of GM-CSF, IFNγ, IL-1α, IL-1β, IL-2, IL-4, IL-7, IL-9, IL-10, IL-12p40, IL-12p70, IL-15, IL-17, IP-10, MIP-2, RANTES or TNF-α was observed at six hours post-injection of alum or IL-33 compared to PBS treated mice (data not shown). Based on the similarity of these cytokine response profiles, we investigated whether the alum-induced increases were mediated by IL-33. To that end, we injected alum in IL-33 KO mice or neutralized IL-33 activity in WT mice using ST2-Fc. Results from these studies showed that of the alum-induced cytokines in WT mice, IL-5, IL-13, MIP-1α and MIP-1β were completely dependent on IL-33 expression while the increase in IL-6, G-CSF, MCP-1 and KC were partially dependent on IL-33 (Fig. 3 and data not shown). IL-33 injection in IL-33 KO mice produced a similar cytokine response as observed in WT mice (data not shown). These results demonstrate that many alum-induced cytokine increases are mediated by IL-33.

### IL-33 injection produces antigen-specific antibody responses

**Figure 4:**
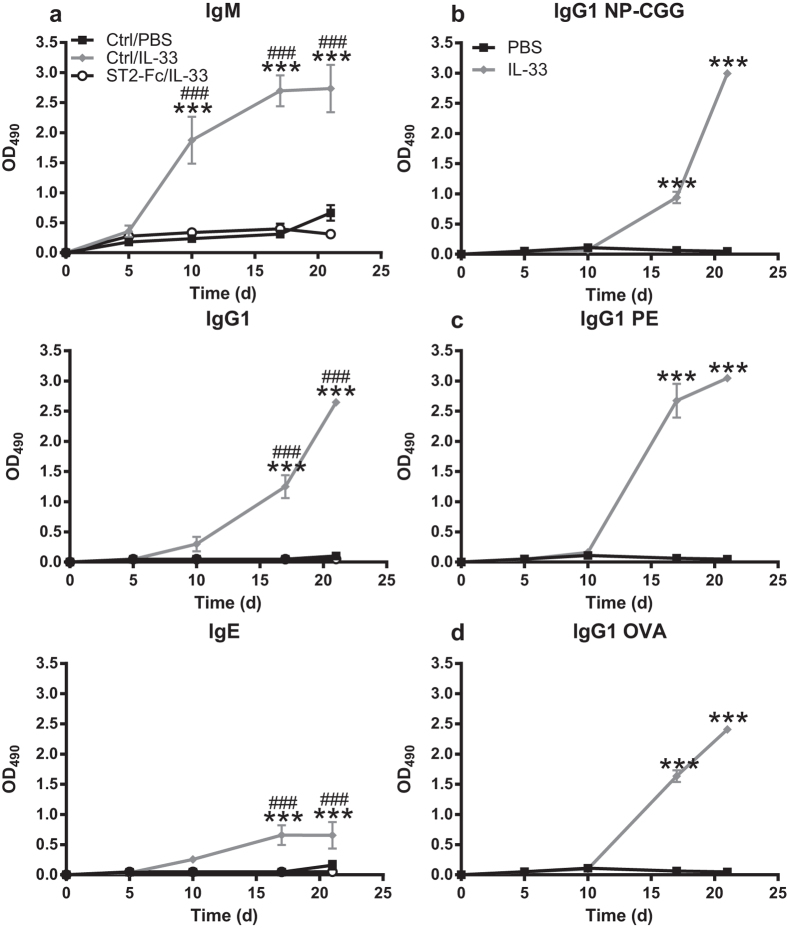
IL-33 injection produces antigen specific antibody responses. (**a**) C57BL/6 mice (n = 5 mice/group) were pre-treated with mouse IgG1 isotype control antibody (Ctrl) or ST2-Fc injected i.p. one hour prior to i.p. injection of PBS/NP-CGG or IL-33/NP-CGG then boosted i.p. on day 14 with PBS/NP-CGG. (b) BALB/c mice (n = 5 mice/group) were injected i.p. with PBS/NP-CGG or IL-33/NP-CGG then boosted i.p. on day 14 with PBS/NP-CGG. (**c**) Mice were treated as in (**b**) except PE was used as the antigen. (**d**) Mice were treated as in (**b**) except OVA was used as the antigen. Antigen specific IgM, IgG1 and IgE serum titers were quantified via ELISA. Data in (**a**) are representative of four independent experiments and data in (**b–d**) are from one experiment. ***p < 0.001 PBS compared to Ctrl/IL-33 groups (Two-way ANOVA with Bonferroni multiple comparison test). ###*p* < 0.001 Ctrl/IL-33 compared to ST2-Fc/IL-33 groups (Two-way ANOVA with Bonferroni multiple comparison test).

Given the similarities between cytokine patterns induced by alum and IL-33 (Fig. 2), we hypothesized that IL-33 itself could serve as an adjuvant. Mice were immunized with a single injection of IL-33/4-Hydroxy-3-nitrophenylacetyl hapten conjugated to chicken gamma globulin at a 30-39 ratio (NP-CGG) or PBS/NP-CGG, received a PBS/NP-CGG boost on day 14 and serum antibody titers were quantified on days 5, 10, 17 and 21 post-immunization. Mice injected with NP-CGG in the presence of IL-33 showed significantly increased NP-specific IgM titers on day 10 which were absent in control mice (Fig. 4a). Following antigen boost, the IgM titers continued to increase on days 17 and 21. Interestingly, little to no NP-specific IgG1 antibodies (the predominant antigen-specific IgG isotype produced with NP-CGG (data not shown)), were observed during the primary response on days 5 or 10 post-immunization. However, IgG1 titers significantly increased on days 17 and 21 following an antigen boost. IL-13 is a critical mediator of IgE production^30^ and IL-33 injection induced release of IL-13 in mice (Fig. 2), therefore we also quantified IgE titers. NP-specific IgE antibody response kinetics showed no significant increases on day 10 but boosting resulted in a significant increase that plateaued on days 17 and 21. To demonstrate that the observed antigen-specific antibody responses were due to IL-33 activity, mice were treated with ST2-Fc prior to immunization with IL-33/NP-CGG. This resulted in a complete absence of NP-specific antibody titers (Fig. 4a), indicating that IL-33 was solely responsible for the observed humoral responses.

To confirm that the IL-33-induced antibody responses were not antigen or mouse strain specific, we injected BALB/c mice with IL-33 and one of three different antigens. BALB/c mice immunized with IL-33 as the adjuvant showed a similar antibody response kinetic and magnitude for NP-specific IgG1 titers (Fig. 4b) as was observed for C57BL/6 mice (Fig. 4a). BALB/c mice immunized with IL-33 and phycoerythrin (PE) or ovalbumin (OVA) had similar antibody responses consisting of no or minimal antigen-specific IgG1 titers on days 5 and 10 that significantly increased on days 17 and 21 following antigen boost (Fig. 4c,d). IgM and IgE titers showed kinetics comparable to that observed in C57BL/6 for all three tested antigens (data not shown). Collectively, we show that a single injection of antigen in conjunction with IL-33 is sufficient to induce a robust antigen-specific antibody response.

### IL-33 mediates different antibody response kinetics than alum

**Figure 5:**
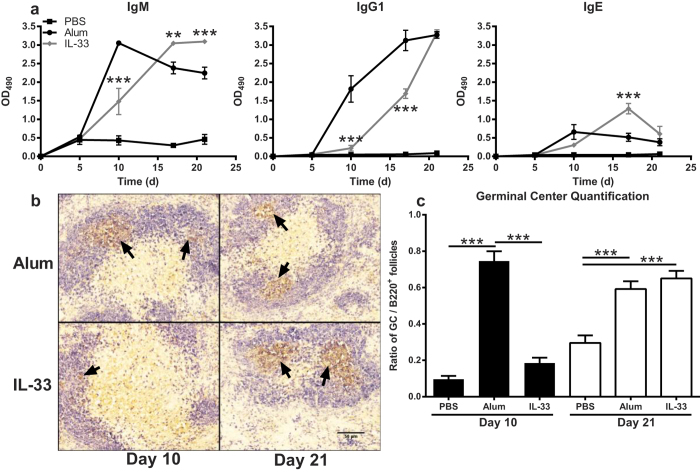
IL-33 mediates different antibody response kinetics than alum. (**a**) C57BL/6 mice (n = 5 mice/group) were injected i.p. with PBS, alum mixed with PBS at 1:2 ratio, or IL-33 in combination with NP-CGG then boosted i.p. on day 14 with PBS/NP-CGG. Antigen specific IgM, IgG1 and IgE serum titers were quantified via ELISA. Data are representative of four independent experiments. **p < 0.01 and ***p < 0.001 IL-33 compared to alum groups (Two-way ANOVA with Bonferroni multiple comparison test). (**b**) Spleens were harvested on days 10 and 21 post-immunization from mice treated as in (**a**). Representative brightfield images (200x magnification) of B220^+^ follicles (blue) and GCs (brown; indicated with black arrows) were captured from stained cryopreserved sections of spleens from each group. (**c**) The numbers of GC and B220^+^ follicles were quantified from sections (n = 8 sections/mouse and 5 mice/group) in (**b**) to calculate the ratio of GCs per B220^+^ follicle. Data are representative of at least two independent experiments. ***p < 0.001 (One-way ANOVA with Tukey’s multiple comparison test).

To evaluate the potential role of IL-33 in driving the adjuvant activity of alum we compared the kinetic antibody response to NP induced by either IL-33 or alum. Both alum and IL-33 injected mice showed significantly increased IgM titers on day 5 compared to PBS controls; however the alum-induced IgM titers were significantly greater than the IL-33-induced titers (Fig. 5a). Following antigen boost, IgM titers in the IL-33 injected mice continued to increase on days 17 and 21, while they decreased in alum injected mice. Although IgG1 titers induced by alum were significantly greater than those induced by IL-33 on days 10 and 17, by day 21 the IL-33-induced IgG1 titers reached the level of those induced by alum. The kinetics of the IgE response was similar to IgM. Alum-induced IgE responses peaked on day 10 while IL-33-induced responses peaked on day 17 following the boost. The results indicate that IL-33 induced a delayed antibody response profile compared to alum during primary and secondary response.

To further investigate the observed differences in the antibody response kinetics between alum and IL-33, we quantified germinal centers (GC) in spleens collected from mice following primary (day 10) and secondary (day 21) immune response via immunohistochemistry. GC from IL-33 injected mice during the primary response were sparse and reduced in size compared to alum injected mice, however the GC reaction observed during the secondary response in IL-33 injected mice was very similar to the primary alum-induced response (Fig. 5b). Preliminary data showed that IL-33 induced significant GC formation in mesenteric lymph nodes by day 4 which was comparable to that induced by alum (data not shown), thus providing a rationale for the observed secondary response. Alum injected mice showed significantly increased numbers of GC per B cell follicle compared to IL-33 injected or PBS control mice on day 10 (Fig. 5c). By day 21, the IL-33 injected mice had a similar number of GC per B cell follicle as the alum-injected mice and both were significantly increased compared to PBS control mice. Thus, the GC formation during the primary and secondary response to immunization with IL-33 as the adjuvant is consistent with the observed kinetics in antigen-specific antibody titers.

**Figure 6:**
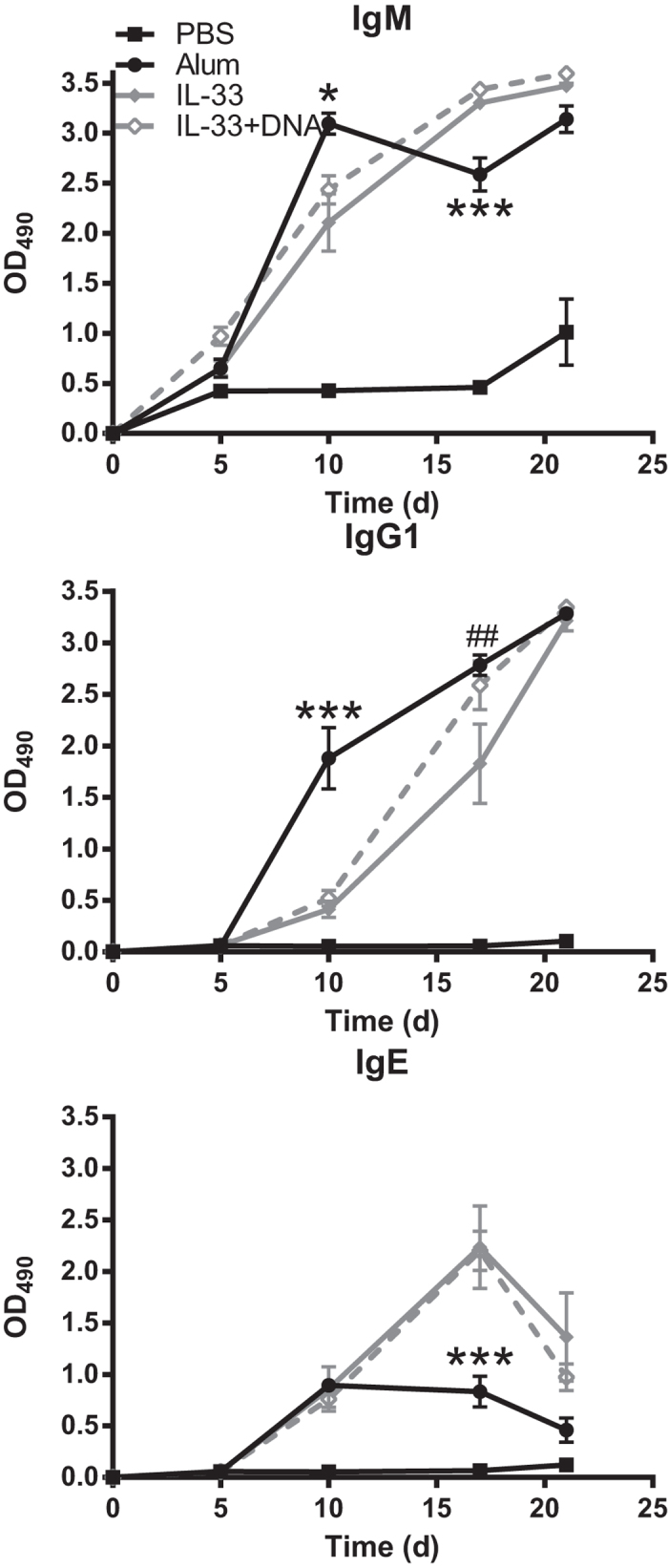
Addition of DNA did not alter IL-33-induced antibody responses. C57BL/6 mice (n = 5 mice/group) were injected i.p. with PBS, alum mixed with PBS at 1:2 ratio, IL-33 or IL-33 mixed with 8μg mouse genomic DNA in combination with NP-CGG then boosted i.p. on day 14 with PBS/NP-CGG. Antigen specific IgM, IgG1and IgE serum titers were quantified via ELISA. Data are representative of two independent experiments. *p < 0.05 and ***p < 0.001 alum compared to IL-33/DNA groups (Two-way ANOVA with Bonferroni multiple comparison test). ##*p* < 0.01 IL-33 compared to IL-33/DNA groups (Two-way ANOVA with Bonferroni multiple comparison test).

In addition to alum-induced release of IL-33 via cellular necrosis (Fig. 1a), other endogenous danger signals also could contribute to the adjuvant activity of alum. Marichal *et al.* showed that alum induced the release of DNA via cellular necrosis and that this was in part responsible for mediating alum adjuvant properties^19^. Since we showed that IL-33 only recapitulates parts of the effects induced by alum, we tested whether co-injection of mouse genomic DNA (to mimic DNA released from necrotic cells) with IL-33/NP-CGG would result in antibody response profiles that were similar to alum-induced responses. Addition of DNA to IL-33 did not appreciably change the IgM, IgG1 or IgE responses relative to IL-33 alone (Fig. 6). A single noted difference was a significant increase in the IgG1 titer on day 17 for the IL-33/DNA group compared to IL-33 alone. These results demonstrate that addition of DNA to IL-33 during immunizations did not enhance the antibody response to levels equivalent to those observed with alum. Therefore, other endogenous danger signals are likely necessary for mediating the full adjuvant activity of alum.

### Alum induces antibody responses independently of IL-33

**Figure 7:**
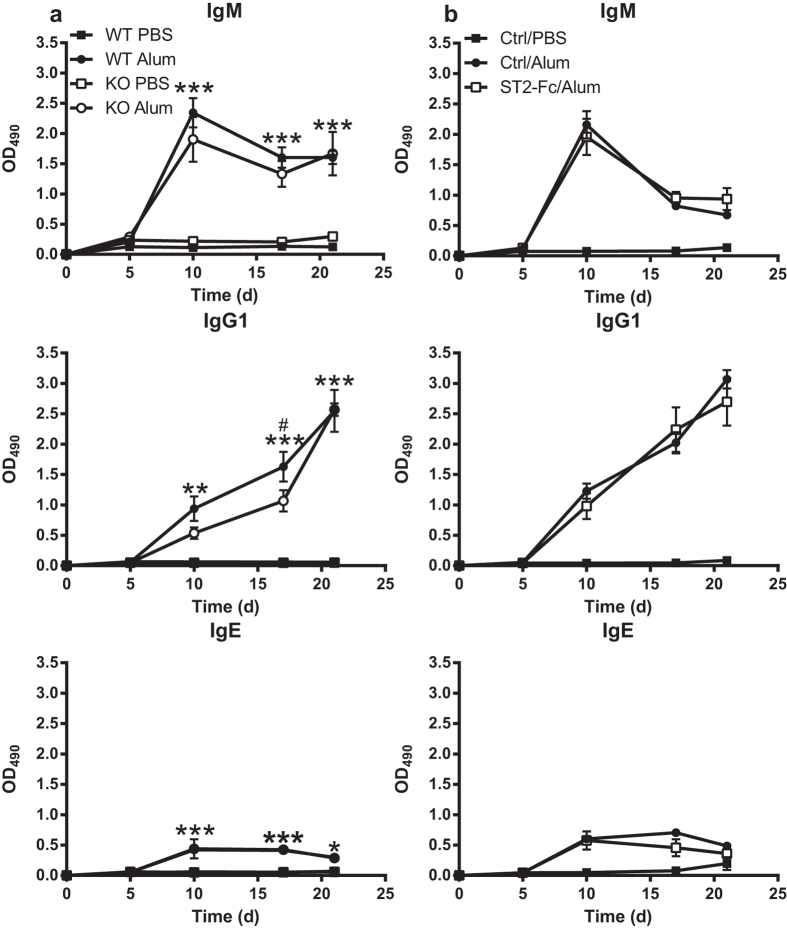
Alum induces antibody responses independently of IL-33. (**a**) WT and IL-33 KO mice (n = 5 mice/group) were injected i.p. with PBS or alum mixed with PBS at 1:2 ratio in combination with NP-CGG then boosted i.p. on day 14 with PBS/NP-CGG. (**b**) C57BL/6 mice (n = 5 mice/group) were pre-treated with mouse IgG1 isotype control antibody (Ctrl) or ST2-Fc injected i.p. one hour prior to i.p. injection of PBS or alum/NP-CGG then boosted on day 14 with PBS/NP-CGG injected i.p. Antigen specific IgM, IgG and IgE serum titers were quantified via ELISA. Data are representative of at least two independent experiments. *p < 0.05 and **p < 0.01 and ***p < 0.001 KO PBS compared to KO alum groups (Two-way ANOVA with Bonferroni multiple comparison test). #*p* < 0.05 WT alum compared to KO alum groups (Two-way ANOVA with Bonferroni multiple comparison test).

To investigate if the presence of IL-33 is necessary for the alum-induced antibody responses, WT and IL-33 KO mice were immunized with alum/NP-CGG. Both WT and IL-33 KO mice showed significantly increased antibody titers compared the PBS/NP-CGG control mice (Fig. 7a). Moreover, alum induced very similar antibody response kinetics for IgM, IgG1 and IgE in WT and IL-33 KO mice, and only IgG1 titers on day 17 in the IL-33 KO mice were significantly lower than WT mice. When WT mice were pre-treated with ST2-Fc prior to immunization with alum/NP-CGG, we did not observe any significant changes in IgM, IgG1 and IgE antibody responses (Fig. 7b), consistent with data observed in the IL-33 KO mice (Fig. 7a). While IL-33 was able to induce cytokine release and a robust secondary antibody response, these results show that alum-induced antibody responses can occur independently of IL-33.

## Discussion

Despite the significant amount of research since the discovery of alum as an adjuvant over 80 years ago, the precise mechanism behind its immunostimulatory properties remains elusive. Recent research showed that alum-induced cellular necrosis results in the release of endogenous danger signals, induction of a T_H_2 cytokine response and recruitment of immune cells that ultimately result in enhanced humoral immune responses^10^,^17^,^19^. Here we show that IL-33 is released following alum-induced localized cellular damage and is directly responsible for several of the alum-induced cytokine increases. We also show that IL-33 exhibits adjuvant-like properties when delivered with an antigen in mice. However, the IL-33-induced antibody response kinetics differ from those induced by alum and are not enhanced with the inclusion of DNA. Using IL-33 KO mice or an IL-33-neutralizing recombinant ST2 receptor, we show that IL-33 is not necessary for the alum-induced humoral responses. Together, these results support a role for IL-33 in the early cytokine response following alum injection but also demonstrate a redundancy in the way alum induces antibody responses.

Based on our data in the IL-33 KO mice and results in other KO mice, alum-induced humoral responses likely are mediated through several redundant systems. For example, IL-5 KO mice injected with alum produce similar IgG1 and IgE titers as WT mice^10^,^31^, while IL-13 KO mice show a defect in IgE responses but not IgG1 responses^32^. Use of NLRP3 or caspase-1 KO mice showed that uric acid-mediated induction of NLRP3 is an important component of the alum-induced IgG1 and IgE response^16^,^18^, however others have presented conflicting data, showing no impact of NLRP3 or caspase-1 deficiency on the adjuvant activity of alum^10^,^19^. Marichal *et al.* also demonstrated that neutralization of released DNA or prevention of DNA-mediated signaling using KO mice significantly inhibited alum-induced IgE titers but had minimal to no impact on IgM or IgG1 titers^19^. Here we show that addition of DNA did not enhance the IL-33-mediated antibody response indicating a potential redundancy in endogenous danger signals. Our data shows that IL-33 potentially is one of several redundant components necessary to mediate the alum-induced antibody response kinetics. Collectively, these results indicate that the adjuvant activity of alum is likely a much more complex interaction of cytokines and endogenous danger signals than previously appreciated.

While our data clearly demonstrate that the early cytokine response induced by alum depends on IL-33, it appears that these cytokines are not necessary to induce a robust antibody response. In support of this, we show that there is no difference in the humoral response between WT and IL-33 KO mice immunized with NP-CGG and alum. Additionally, mice lacking MyD88, an important signaling adaptor for ST2, show no difference in IgM, IgG1 or IgE antibody responses following alum injection^33^. This also is consistent with the observation that nodules induced by alum injection are not necessary for adjuvant activity^6^,^7^. Munks *et al.* proposed that alum injection likely results in a foreign body response that occurs independently of the alum-induced antibody response^6^. While other have shown that IL-33 exhibits some adjuvant activity they tested multiple injections with IL-33 and only showed antibody titers at a single time point 42 days after the first immunization^34^,^35^. We show that a single injection of IL-33 with an antigen followed by an antigen-only boost induces a robust secondary antibody response which is comparable to that induced by alum. Therefore, IL-33 potentially could act as an adjuvant that induces a similar innate immune response as alum but independently mediates a differential type and/or quality of humoral response resulting in differing antibody response kinetics.

Cellular immune responses also are an important component of alum-mediated adjuvant activity^2^,^6^,^7^,^10^,^13^. Although our data focuses on the impact of IL-33 on the humoral immune response, data from others show that IL-33 also is involved in the cellular immune response. Villarreal *et al.* showed that co-injection of IL-33 with a DNA vaccine induces a potent antigen specific effector and memory T cell immunity in a mouse tumor model^36^. Transgenic expression of IL-33 in mouse tumor models enhances cytotoxic activity of CD8^+^ T cells and NK cells^37^. A potential mechanism for these observed outcomes is that CD8^+^ T cells express ST2 and IL-33 can synergize with T cell receptor and IL-12 signaling to induce production of IFNγ^38^. Bonilla *et al.* also showed that IL-33 KO mice produce significantly less lymphocytic choriomeningitis virus-specific cytotoxic T lymphocytes following infection and the protective effects of IL-33 were mediated via MyD88 signaling and release of IFNγ, TNFα, and other inflammatory signals^39^. Additionally, T_H_2 cells likely are involved in T cell mediated immunity as IL-33 can induce naïve CD4^+^ T cells to differentiate into IL-5 producing T_H_2 cells independently of IL-4^40^. IL-33-induced differentation of T cells is mediated via dendritic cells as only co-cultures of bone marrow-derived dendritic cells and naïve CD4^+^ T cells results in production of IL-5^41^,^42^. Consequently, IL-33-mediated adjuvant activity likely is a combination of cellular and humoral immune responses that depend on the local environment in which IL-33 is released following cellular damage. Therefore, further studies on the role of IL-33 in mediating cellular immune responses following immunization at different anatomical sites could provide additional insights into the adjuvant-like properties of IL-33.

In summary, we show that injection of alum produces localized cellular necrosis resulting in the release of IL-33 which mediates a T_H_2- and inflammation-associated cytokine response. IL-33 alone is capable of inducing a robust secondary antibody response but is not necessary for the alum-induced humoral response. Further research is necessary to understand how IL-33 is driving such a secondary response. Collectively, these results provide novel insights into the mechanism of action for alum-mediated adjuvant activity. Improved understanding of alum immunostimulatory properties can result in the development of novel adjuvant combinations that selectively enhance different aspect of the immune response and enable new types of vaccines.

## Materials and Methods

### Mice

WT C57BL/6 mice were obtained from Taconic. IL-33 KO mice on a C57BL/6 background were obtained from the Knockout Mouse Project (KOMP) Repository (www.komp.org) and custom bred at Taconic. BALB/c mice were purchased from Jackson Laboratories. All animals were housed and maintained at Eli Lilly and Company (Indianapolis, IN). All animal experiments were performed in accordance with the research guidelines approved by the Eli Lilly and Company Institutional Animal Care and Use Committee.

### Peritoneal lavage and cell viability measurement

WT or IL-33 KO mice were injected i.p. with 100 μL/mouse PBS (Thermo Fisher Scientific) or Alhydrogel 2% (alum; Invivogen) mixed with PBS at a 1:2 ratio (0.3 mg alum/mouse). After thirty minutes, the peritoneal cavity was lavaged with 3 mL of PBS. Cells were pelleted by centrifugation and the acellular fraction was collected into separate tubes. The percentage of necrotic cells was quantified using Guava ViaCount solution (Millipore) and the Guava ViaCount function on a Guava EasyCyte Plus (Millipore). This assay uses two DNA binding dyes to differentiate between viable, apoptotic, and dead cells. The acellular fraction was concentrated 20-fold using Amicon Ultracel 3K tubes (Millipore) by following the manufacturer’s instructions. IL-33 levels were quantified using a mouse IL-33 ELISA (R&D Systems).

### *In vitro* splenocyte viability assay

Splenocytes were isolated from WT mice and cultured (1 × 10^5^ cells/well) in a 96 well plate (Corning) in RPMI (Life Technologies) with 5% heat inactivated fetal bovine serum (Life Technologies) and 1% antibiotic/antimycotic (HyClone). PBS (100 μL) or alum mixed with PBS at 1:2 ratio (100 μL) was added for selected times then cellular necrosis was quantified using Guava ViaCount. IL-33 was quantified in culture supernatants via ELISA.

### *In vivo* induction of cytokines

WT or IL-33 KO mice were injected i.p. with 100 μL of PBS, alum (1 mg/mouse), alum mixed with PBS at 1:1 (0.5 mg/mouse ), 1:2 (0.3 mg/mouse) or 1:5 (0.2 mg/mouse) ratios or 500 ng/mouse, 50 ng/mouse, or 5 ng/mouse mature (aa102–266) form recombinant murine IL-33 (produced in-house). In some experiments, mice were injected i.p. with 100 μg/mouse of mouse IgG1 isotype control antibody or 100 μg/mouse of recombinant soluble murine ST2 fused to murine IgG1 Fc domain (ST2-Fc; both produced in-house) one hour prior to alum injections. Serum was collected six hours post-alum or IL-33 injection and cytokine levels were quantified using a Millipore Milliplex Mouse Cytokine/Chemokine Magnetic Bead Panel (G-CSF, GM-CSF, IFNγ, IL-1α, IL-1β, IL-2, IL-4, IL-5, Il-6, IL-7, IL-9, IL-10, IL-12p40, IL-12p70, IL-13, IL-15, IL-17, IP-10, KC, MCP-1, MIP-1α, MIP-1β, MIP-2, RANTES, TNF-α) on the Luminex 200 instrument according to the manufacturer’s instructions.

### Immunizations

WT and IL-33 KO mice were immunized i.p. with 2 μg/mouse of NP-CGG (Biosearch Technologies) in PBS as a non-adjuvant control (PBS/NP-CGG), in a 1:2 alum:PBS mix (alum/NP-CGG), or with 50 ng/mouse IL-33 (IL-33/NP-CGG). For IL-33 neutralization experiments, mice were injected i.p. with 100 μg/mouse of mouse IgG1 isotype control antibody or ST2-Fc one hour prior to immunization. For DNA adjuvant experiments, mice were injected i.p. with 8 μg/mouse of mouse genomic DNA (Promega) and IL-33/NP-CGG. BALB/c mice were injected i.p. with PBS/NP-CGG, IL-33/NP-CGG, or 50 ng/mouse of IL-33 in combination with 10 μg/mouse PE (Anaspec) or 50 μg/mouse Grade VI OVA (Sigma). All WT, KO, or BALB/c mice were boosted on day 14 post-immunization with PBS/NP-CGG. Blood was collected into serum separator microtainers (BD) on days 5, 10, 17 and 21 post-immunization. Serum was collected and stored at −80 ^°^C until analysis.

### Antigen specific-antibody ELISA

Based on the antigen used for immunizations, NP conjugated to BSA at a >20 ratio (Biosearch Technologies), PE or OVA all diluted in PBS were coated onto separate 96 well plates (Greiner Bio-one) at 2 μg/mL and incubated overnight at 4 ^°^C. Plates were washed then blocked with casein (Thermo Scientific) for one hour at room temperature (RT). Serum samples were serially diluted in casein then added to the plates and incubated for two hours at RT. After washing, biotin rat anti-mouse IgG1 (BD), peroxidase conjugated goat anti-mouse IgM (Jackson Immunoresearch) or biotin rat anti-mouse IgE (BD) was added to the plates and incubated for one hour at RT. Plates were washed and streptavidin HRP (Jackson Immunoresearch) was added to the IgG1 and IgE plates and incubated for thirty minutes at RT. Plates were washed then developed for twenty minutes with an o-Phenylenediamine dihydrochloride tablet (Sigma) dissolved in 100 mM Sodium phosphate dibasic (Mallinckrodt), 50 mM citric acid (Mallinckrodt) and 0.04% hydrogen peroxide (Mallinckrodt) at pH 5.0. Reactions were stopped using 1N HCL (Fisher Scientific) and plates were read at 490λ using a Molecular Devices Spectra Max 340 PC running Soft Max Pro 3.1.2. All antibody data are presented at IgM (1:1000), IgG1 (1:80,000), and IgE (1:100) serum dilutions which represent non-saturating concentrations.

### Immunohistochemistry of spleens and germinal center quantification

For analysis of GC formation, spleens were collected at 10 and 21 days post-immunization from mice injected with PBS/NP-CGG, alum/NP-CGG, or IL-33/NP-CGG. The spleens were fixed in 1.6% paraformaldehyde (EMS) and 20% sucrose (Sigma) overnight at 4 ^°^C, embedded in optimal cutting temperature medium (Sakura), snap frozen on top of liquid nitrogen and stored at −80 ^°^C. Spleen sections (8 μm) were cut using a Leica CM3050S Cryostat and adhered to Superfrost Plus Slides (Fisher Scientific). Slides were rinsed in PBS then incubated with Dual Endogenous Enzyme block (Dako) for ten minutes at RT. After rinsing in PBS slides were incubated with avidin and biotin blocking reagents (Vector Laboratories) according to the manufacturer’s instructions. Slides were washed and blocked with 10% BSA/PBS (Life Technologies) for twenty minutes at RT. Blocking buffer was removed and biotin-PNA (Vector Laboratories) at 50 μg/mL and rat anti-mouse B220 (BD) at 2.5 μg/mL diluted in blocking buffer were added to the slides and incubated overnight at 4 ^°^C. Slides were washed in PBS and streptavidin-horseradish peroxidase (Covance) and alkaline phosphatase-conjugated goat anti-rat IgG (Jackson Immunoresearch) at 2.4 μg/mL were added to the slides and incubated for thirty minutes at RT. Slides were washed in PBS and incubated with BCIP/NBT AP Substrate (Vector Laboratories) followed by 3, 3′-Diaminobenzidine (Covance) both according to manufacturer’s instructions. The slides were rinsed in PBS, dehydrated with ethanol (Sigma), cleared with xylenes (Sigma) and cover slipped using Cytoseal (Thermo Fisher Scientific). Images were captured at 200x magnification on a Nikon Eclipse 80i microscope. Total GC, identified as PNA^+^ cell aggregates, and B220^+^ follicles, identified as discrete B220^+^ cell clusters, per spleen were manually enumerated to calculate the ratio of GC per B220^+^ follicle for each spleen from mice within the different immunization groups.

### Statistical analysis

Statistical analyses were performed using GraphPad Prism software (version 6) and all graphs show mean ± SEM. A *p* value < 0.05 was considered significant.

## Additional Information

**How to cite this article**: Rose, W. A. *et al.* IL-33 released by alum is responsible for early cytokine production and has adjuvant properties. *Sci. Rep.* **5**, 13146; doi: 10.1038/srep13146 (2015).
